# Factors influencing abandoned farmland in hilly and mountainous areas, and the governance paths: A case study of Xingning City

**DOI:** 10.1371/journal.pone.0271498

**Published:** 2022-07-18

**Authors:** Minghua Wu, Guangsheng Liu, Siyang She, Lesong Zhao

**Affiliations:** 1 College of Water Conservancy and Civil Engineering, South China Agricultural University, Guangzhou, Guangdong, China; 2 Guangdong Province Key Laboratory of Land Use and Consolidation, Guangzhou, Guangdong, China; 3 College of Public Administration, South China Agricultural University, Guangzhou, Guangdong, China; Northeastern University (Shenyang China), CHINA

## Abstract

The current global pandemic has laid bare the importance of national food security to human survival. Many cultivated lands in the hilly, mountainous, and other marginalized areas have been abandoned on a large scale, resulting in a tremendous waste of agricultural resources, thereby threatening national food security. Here, we studied abandoned farmland in Xingning City, a mountainous area in northern Guangdong province. According to the "seeding—growing—harvesting" life cycle of cultivated plots, spatial superposition method and remote sensing change detection method were applied to identify abandoned arable land. Logistic regression model was used to reveal the influencing factors and occurrence mechanism of abandoned cropland at plot scale, and cluster analysis was used to discuss the classification and management strategies. Result showed that 16.83% of the cultivated land in the study area was severely abandoned, attributed to poor location, poor basic conditions, and fragmentation of the land. Further, the abandoned farmland was divided into output-driving type, cultivation condition-driving type, and plot-condition driving type. Based on these types, we proposed some countermeasures, such as adjusting agricultural structures, tamping agricultural infrastructures, strengthening land circulation, popularizing appropriate scale operations. These measures provide a reference to effectively curb abandoned farmland and improving the utilization efficiency of cultivated land, especially in recent years.

## Introduction

Land cultivation is the basic agricultural practice that guarantees human survival. Abandoned cultivated land refers to cultivated land that has been idle or barren for a certain period. Because of the rapid urbanization and industrialization, the large-scale rural labor transfer to cities and towns has led to high cropland abandonment in marginalized areas, such as mountains and hills [1]. Increasing cropland abandonment wastes valuable cropland resources, reduces grain output, and degrades agricultural facilities, and reduces the effectiveness of national farmland improvement and development inputs [2, 3]. China’s per capita arable land is less than 1.5 mu, causing a food supply-demand imbalance [4]. The country’s annual imports of grain crops are equivalent to 800 million mu of arable land sown, and by 2030, there will be a shortfall of about 31 million tons of grain. In the face of the pressure of national red line protection of cultivated land and people’s livelihood demands to ensure food security, how to reduce the waste of cultivated land caused by abandoned land to achieve the goal of land conservation and intensive use proposed by the land management department is the key and difficult point of sustainable land use management and cultivated land protection in China at present and even in the future [5].

Cropland abandonment is an important research aspect of land use and land cover change. Judging from the current literature, scholars had conducted a lot of discussion and analysis on the driving force and mechanism of the cropland abandonment, the spatial distribution characteristics and influencing factors (determining factors) of the cropland abandonment, the ecological environment and social effects of the cropland abandonment, and the policies to deal with the cropland abandonment. The focus of this article is on the driving mechanism of cropland abandonment. Therefore, this article will focus on reviewing the research on the driving mechanism of cropland abandonment. Cropland abandonment results from a comprehensive impact of multiple factors [6], including macro-scale socio-economic factors, household-scale factors, and plot factors (such as physical geography and farming conditions). Macro-level socio-economic factors are the main driving force behind the abandonment of cultivated land. In summary, the macro-social and economic factors of farmland abandonment mainly include population migration, relatively low agricultural returns, and land system reforms. Benayas et al. [7] opined that socio-economic factors are more important than natural ecological factors. Elsewhere, the abandoned farmland in Poland, Slovakia, and Ukraine were compared. It was found that although natural topographical factors impact the abandoned farmland, it is more affected by the land system, land reform policy, and rural population density [8]. In the same year, Preshchepov et al. [9] analyzed the factors that influenced Russia’s cropland abandonment from 1990 to 2000. They inferred that the social factors (such as grain production and per capita density factors) impact cropland abandonment more than any other factor. Later, Liu et al. [10] reported that lower agricultural returns are the key reason for cropland abandonment. Deininger et al. [11] concluded that the solidification of land contract rights and unclear land ownership increase transaction costs, hinder the development of the farmland transfer market, and promote cropland abandonment. Before then, Tan [12] had stated that in the absence of a rural transfer market, migrant labor could not be allocated to farmers with relatively abundant labor force through the transfer, thereby promoting cropland abandonment.

Farmer households are the decision-making unit for the use of farmland, and farmland abandonment is the result of farmer households’ allocation of labor. Different types of farmers have different treatment methods for marginal land, and the reasons, degrees and influencing factors of abandonment are also different. Farmer-scale research is an important perspective for the study of abandonment mechanism. He et al. [13] used behaviour decision models of farming households to systematically understand and analysed the behavioural mechanism leading to cropland abandonment by different types of farming households, including aged households, stable part-time households, unstable part-time households and pure households. Wang et al. [14] used a variety of metrics and binary Logit model to conduct a two-stage tracking survey involving 7045 plots from 1012 households from the southwest mountainous areas of China in 2011 and 2018. Li et al. [15] analyzes the difference on farmers’ behaviors of cultivated land abandonment based on household survey and land plot survey in 12 typical villages of Chongqing, and explores of the factors influencing the cultivated land abandonment by adopting multivariate linear regression model.

From a microscopic point of view, within the same landform unit, the risk and degree of abandonment of cultivated land between different plots are different. Therefore, the microscopic plot factors that affect agricultural input costs and output benefits are also important factors influencing the abandonment of cultivated land. Liang et al. [16] investigated regional distribution features of sloping farmland and abandoned farmland in the hinterland of the Three Gorges Reservoir Area (TGRA) using high-resolution remote sensing images, ArcGIS spatial analysis, and statistical methods. Moreso, a negative correlation exists between farmland transfer and cropland abandonment in mountainous areas or village level, and that the abandonment rate under superior tillage was significantly lower than that under inferior tillage at plot scale [17–19].

The mechanism of cropland abandonment is complicated, and there are considerable differences between scales. Research on driving factors of cropland abandonment at macro scale takes administrative districts as research unit, and pays more attention to social and economic factors, so it is difficult to deeply reveal the influencing factors at micro level. While the estimates of first-hand data collected from household surveys may be more accurate, but they lack spatial details. The spatial determinants are difficult to identify, so the decision-making support for abandoned farmland is relatively weak [20]. At present, there are more researches on the driving mechanisms of cropland abandonment at the macro scale and household scale, but less researches on the driving factors of cropland abandonment at the plot scale, which still needs to be further strengthened.

Xingning City is a relatively backward, mountainous area of North Guangdong Province, China. Due to geographic restrictions, many rural laborers migrate to more developed regions (such as the Pearl River Delta of South, Guangdong Province, China), causing severe cropland abandonment. With Xingning city as the research area, this paper studied the occurrence mechanism of cropland abandonment at the plot scale by applying landscape ecology and statistical analysis methods. It also explored the countermeasures for cropland abandonment in mountainous areas. This research includes these three aspects: (i) identification and status analysis of abandoned cropland through remote sensing; (ii) identification and mechanism study of the main controlling factors based on a Logistic Model; (iii) classification and management analysis of abandoned cropland via cluster analysis.

Our research results and conclusions can provide decision-making support for the development of abandoned cultivated lands in this region, serving as a reference for other mountainous areas. Eventually, it would help in promoting the implementation of rural revitalization strategies.

## Materials and methods

### The study area

**Fig 1:**
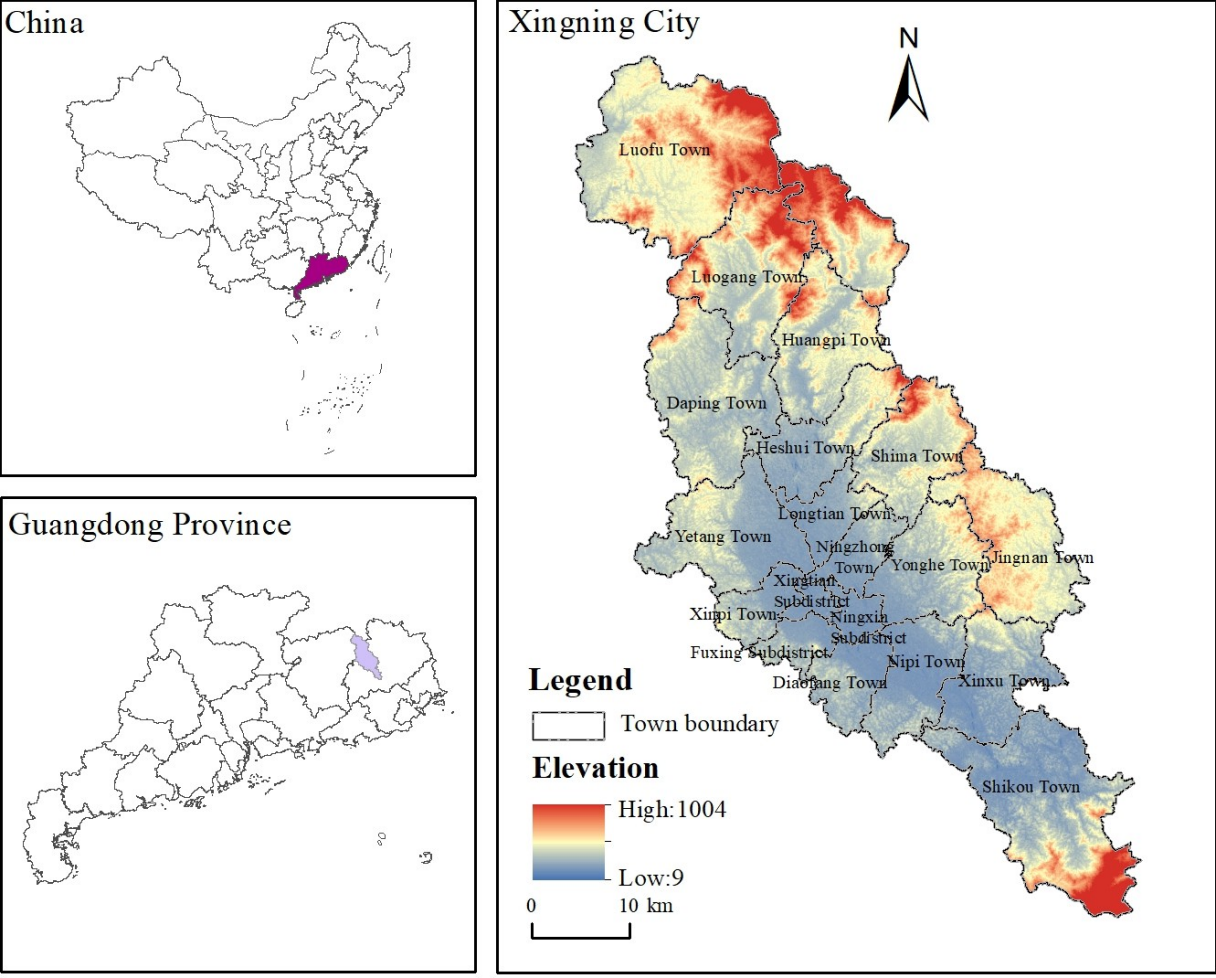
A map showing the geographical location of the study area.

Xingning City is located in the mountainous area of northern Guangdong, China. On the upper reaches of the Dongjiang and Hanjiang Rivers, the city spans 115°30′-116° east longitude and 23°50′-24°37′ north latitude (Fig 1). It is adjacent to Xunwu County in Jiangxi Province in the north, Pingyuan County and Meixian District in Meizhou City in the northeast, Fengshun County and Meixian District in Meizhou City in the south, Longchuan County in the northwest, and Meizhou City in the southwest. Xingning City’s land area is 2075.39 km^2^. Mountains surround the territory, and the central part is a faulted basin of more than 300 km^2^.

The city (county) is flatboat in shape, having three types of plains: platforms with altitudes of <200 m, hills between 200 and 400 m, and mountains >400 m account for 38.1%, 49.69%, and 12.21% of the land area, respectively. Xingning has a transitional climate between South Asia and Mid-subtropical, with an average annual temperature of 20.4°C. The natural environment is serene, and the frost-free period is long. Overall, the climatic conditions favor plant growth, forestry, animal husbandry, fishery, and other industries. It is suitable for growing rice, peanuts, tea and other grains and cash crops. The seventh census data shows that the total population of Xingning City is 7.79×10^5^, including 4.14×10^5^ rural registered population, accounting for 53.08%. The cultivated land area of Xingning is 347.85km^2^, accounting for 16.93% of the total land area, but the per capita cultivated land area is only 0.03hm^2^/person. In recent years, due to the impact of labor migration and the aging of the agricultural population, the phenomenon of cropland abandonment is more common. Therefore, the choice of Xingning City as the research area of cropland abandonment is typical.

### Research data

The research data include the land-use change survey data in Xingning City between 2009 and 2017, Landsat8 (30 m, band number 121, row number 44) remote sensing image data in September and December of 2016 and 2017, Google Earth high-definition image (0.54 m), DEM data (30 m), tillage conditions, and cultivated land quality data. Land-use change survey data and Landsat8 remote sensing image data were used to identify abandoned cultivated land from Xingning Natural Resources Bureau and Geospatial Data Cloud (www.gscloud.cn). Google Earth HD image (0.54 m) was used to verify the accuracy of cropland abandonment, while DEM data helped extract geographical impact factors (such as slope and elevation) derived from geospatial data cloud. Distance factors (such as farming radius) were obtained from the current land use survey data in Xingning City. Data, such as cultivated land output and soil quality, were acquired from the cultivated land quality database of Xingning City.

### Research method and process

#### Abandoned farmland identification

Generally, cultivated land, idle for two consecutive years, can be regarded as abandoned land [12]. This research combines land-use change survey data with remote sensing detection to identify abandoned farmland. After the abandonment of cropland, the land cover types changed from crop to grassland, shrub, and other land cover types. To this end, the identification of abandoned cultivated land is completed in two steps:

*Step 1*: The land-use change data were used to identify the abandoned cropland with land-type change. The change of land type can reflect the abandonment of cropland, showing the emergence of grassland, woodland, and unused land [21]. The cultivated land map plots of the 2015 land-use change survey were taken as the base period. The 2017 land-use change survey data were superimposed to obtain the cultivated land for other land-use types. Then, the cultivated land in 2015 and the grassland and unused map plots in 2017 were selected as the abandoned cultivated land.

**Fig 2:**
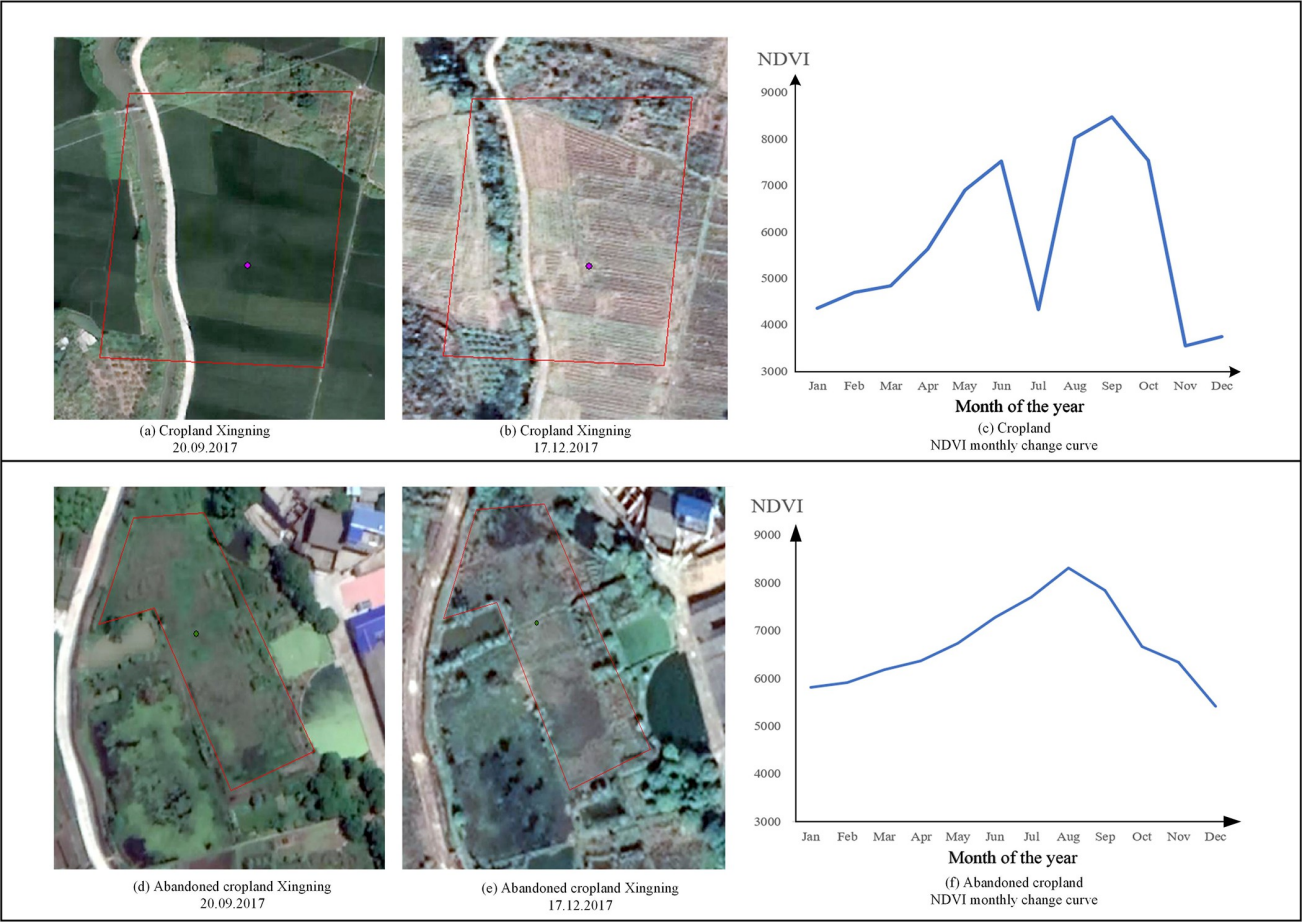
NDVI time series variation curves of cultivated land and abandoned land.

*Step 2*: The uncultivated farmland was identified based on remote sensing images. Abandoned cultivated land obtained through land change survey data was converted in land use [22]. However, it is impossible to identify cultivated land that has not yet been converted, although uncultivated. Due to the growth cycle changes of crops (i.e., seeding, growing, and harvesting) in cultivated plots [23, 24], the change of normalized differential vegetation index (NDVI) is relatively drastic. However, in the short term, the land cover of abandoned farmland is usually bare land or grassland [25], and the change of NDVI value is small [26]. In the current study, NDVI change detection was used to identify cropland abandonment according to the change patterns of the land cover of cultivated and abandoned plots [27]. The following specific method was followed: The cultivated land NDVI values in the growing seasons (September) and the non-growing season (December) were selected (Fig 2), and the gap between the two seasons was calculated [28].

**Fig 3:**
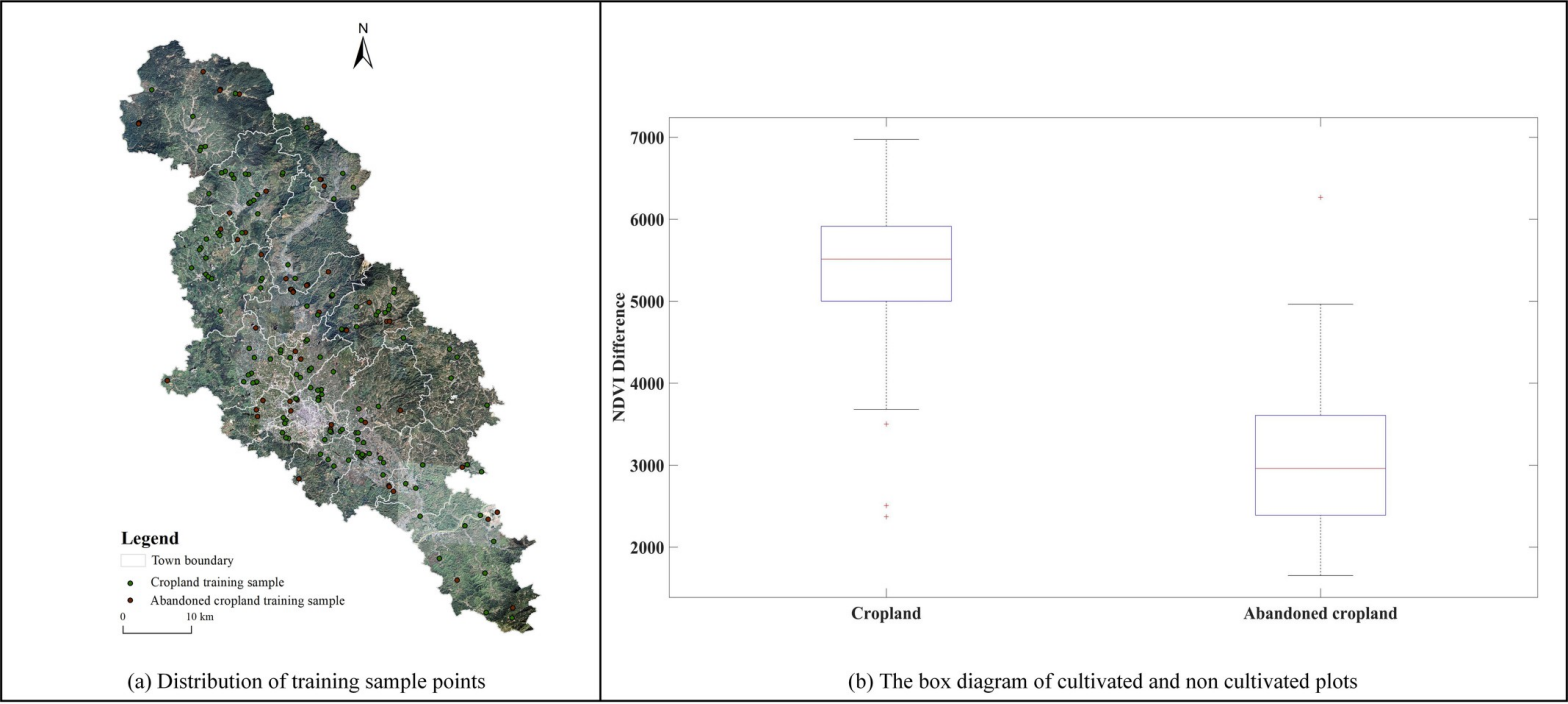
Sample points and box diagram of cultivated plots and non-cultivated plots.

Based on the field survey photos of abandoned cultivated land, 53 abandoned land plots and 113 cultivated land plots were selected as statistical samples. A box chart [29] was drawn to determine the threshold range of NDVI difference between cultivated and abandoned land (Fig 3). Based on the threshold value, the abandoned farmland for two years was continuously extracted, and the intersection was used to obtain the abandoned farmland. Finally, the information was combined with the abandoned farmland identified by the land-change survey to form the final abandoned farmland.

#### Accuracy verification

To verify the accuracy of the abandoned cropland identification, the following steps were followed. First, image plots of abandoned cropland were randomly extracted and compared with Google Earth high-resolution image (resolution 0.54 m). Then, the proportion of the abandoned farmland in the total verification sample was calculated. Afterward, 10% (887 samples) of the total amount of abandoned farmland was randomly selected from the towns (streets) of Xingning as the verification sample. The verification sample chosen was combined with the Google earth high-resolution image. The comprehensive visual judgment verified each abandoned farming map spot (818 identified abandoned farming samples) by ground-truthing. Finally, the rate of abandoned farming map spots was comprehensively calculated, and the accuracy of the map identification was 92% [1].

#### Logistic regression model

This study focused on the occurrence mechanism of cropland abandonment at the microscopic plot scale. In the hilly and mountainous areas, the land resource endowment varies significantly due to the land’s natural quality and the cultivation conditions [30]. The physical geographical factors considered include plot size, cultivated land type, plot shape, slope, elevation, proximity to woodland, soil quality, and cultivated land output. Generally, the possibility of abandonment of dryland in hilly areas is higher than that of paddy fields. Cultivated lands with large plots and regular shapes are easier to operate on a large scale, and the possibility of abandonment lessened. Also, the higher the elevation, the greater the chance of abandonment. Due to the proximity of cultivated land to forestland, it is vulnerable to forest seed rain and wildlife, resulting in low yield of cultivated land and high susceptibility to abandonment. Besides, the worse the soil quality and land output, the higher the likelihood of abandonment.

The farming-limiting factors investigated include farming distance, convenient transportation, and irrigation conditions. Usually, the farther the cropland is to the rural settlements, the higher the farming cost. In the case of scarce labor, the farther the cropland is from the village, the more likely it will be abandoned. The closer the cultivated land is to the road, the lower the transportation cost of crops, the more convenient the cultivation, and the lower the possibility of abandonment. Likewise, the closer the cultivated land is to the ditch, the better the irrigation condition, and the lower the possibility of abandonment [31]. Therefore, the influencing factor system of cropland abandonment at the plot scale was established (Table 1).

**Table 1:** Factors influencing cropland abandonment at the plot scale.

Variable type	Variable name	Index layer	Hypothetical relationship
**Dependent variable (Y)**	Abandoned/not abandoned	Abandoned is marked as 1, and not abandoned is marked as 0	
**Independent variable (X)**	Physical geography factors	Elevation	-
		Slope	-
		Plot size	+
		Plot shape	+
		Soil quality	+
		Cultivated land output	+
		Distance to woodland	-
	Farming condition factors	Tillage distance	-
		Distance to ditch	+
		Distance to road	+

Abandoned lands were marked as 1, while those unabandoned as 0. A logistic regression model (Eq 1) was used to establish the relationship between abandonment and its influencing factors.

$$ln\;\left(\frac{p_{i}}{1-p_{i}}\right)=\alpha +\beta_{0}X_{0}+\beta_{1}X_{1}+\cdots +\beta_{n}X_{n}$$ (1)

where *p*_*i*_ is the conditional probability of abandonment of the sample point; α is a constant term; *X*_*0*_,*…*,*X*_*n*_ are the influencing factors of the occurrence of cropland abandonment; *β*_*0*_, *β*_*1*_,*…*,*β*_*n*_ are the regression coefficients to be obtained. When the coefficient is positive (negative), the corresponding independent variable *X*_*k*_(*k = 1~n*) can increase (decrease) the probability of cropland abandonment occurrence. Also, the higher the coefficient (absolute value), the greater the variable’s effect on cropland abandonment.

## Results

### Distribution pattern of abandoned farmland

**Fig 4:**
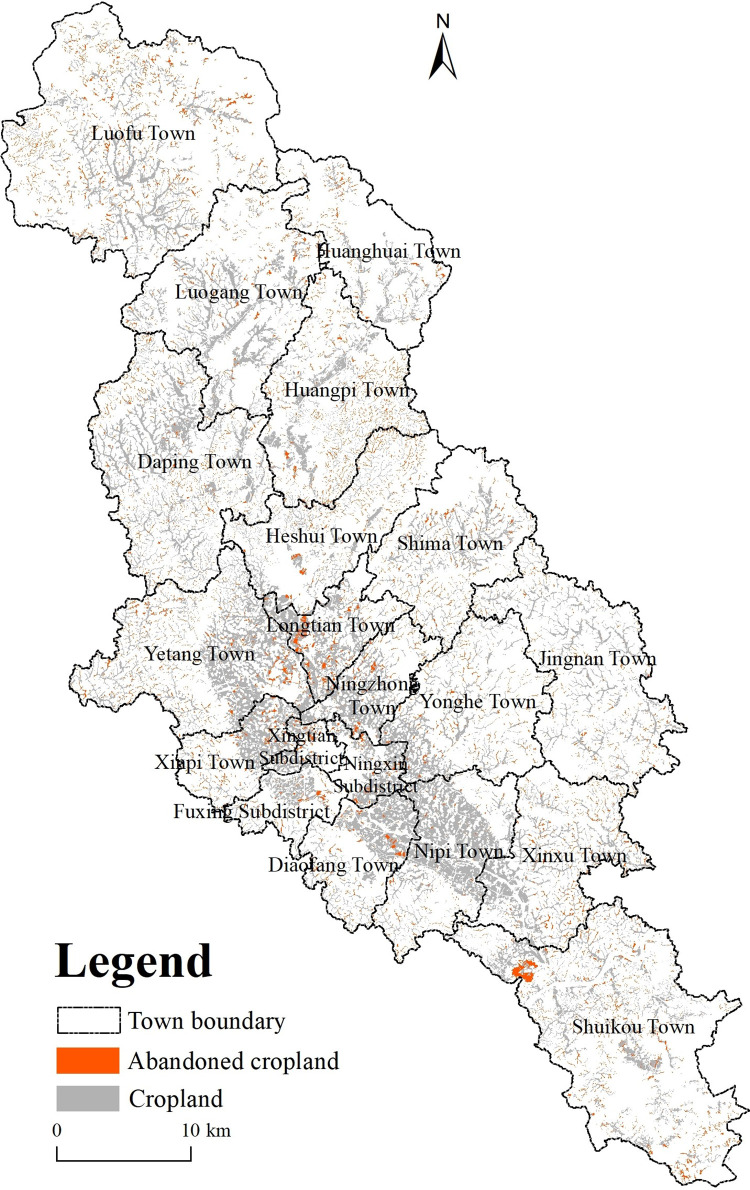
Spatial distribution map of abandoned farmland in the study area.

Table 2 summarized the abandoned area of cropland by town. The abandoned cultivated land area is 5854.36 hm^2^, accounting for 16.83% of the total cultivated land area (Fig 4). Here, the abandonment was relatively severe. Among the types of abandoned cultivated land, the proportion of abandoned dry land (30%) was the largest, and the uncultivated dry land was mainly used for planting fruit trees and tea trees. Next was paddy field abandonment (≈15%), causing a huge waste of cultivated land resources in the study area.

**Table 2:** Statistical table of the characteristics of abandoned cropland.

Variable	Classification	Proportion of cropland abandoned
**Slope (°)**	≤2	8.63%
	(2,5)	24.46%
	(5,10)	33.12%
	(10,15)	19.93%
	(15,25)	12.08%
	>25	1.77%
**parcel area (hm** ^**2**^ **)**	≤1	80.96%
	(1,2)	12.75%
	>2	6.29%
**Distance to woodland (m)**	≤100	73.52%
	(100,200)	11.32%
	(200,500)	11.66%
	(500,1000)	3.30%
	>1000	0.21%
**Distance to the ditch (m)**	≤50	23.20%
	(50,100)	11.10%
	(100,200)	16.43%
	(200,500)	29.34%
	(500,1000)	14.73%
	>1000	5.20%
**Distance to rural road (m)**	≤50	31.98%
	(50,100)	13.90%
	(100,200)	19.67%
	(200,500)	25.21%
	(500,1000)	8.33%
	>1000	0.21%

**Fig 5:**
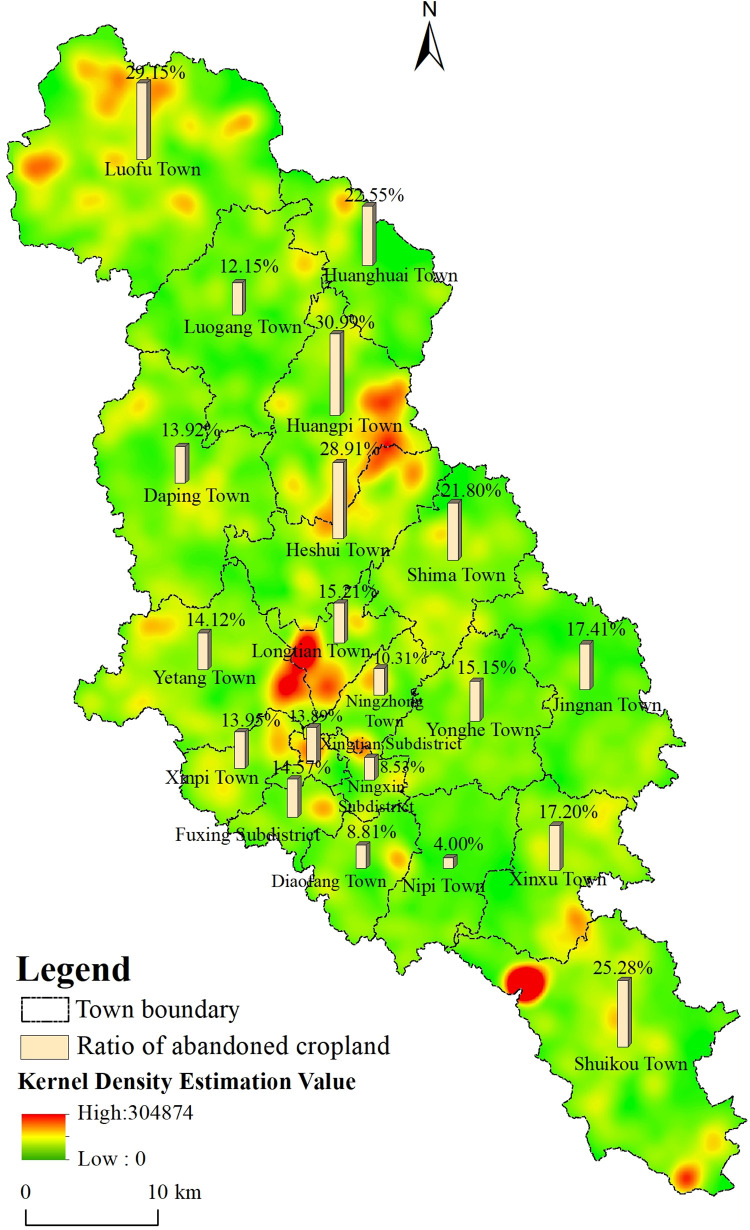
Nuclear density and distribution ratio of abandoned cropland in the study area.

Through spatial distribution analysis, we observed that abandonment of farmland in the periphery of the main urban area was severe, while it was less with farmlands located in the main urban area. For instance, Huangbei town and Luofu town had the largest proportion of abandoned land, accounting for 29.67% and 29.06% of the cultivated land area, respectively. The proportion of abandoned land in Ningzhong town, Ningxin street, Diafang town, and Nibei town was <10%, while that in other towns was between 10% and 20% (Fig 5).

To express the spatial pattern distribution of abandoned cropland, the Kernel Density Estimation (KDE) method converted abandoned cropland into points. The area of abandoned cropland was taken as a field of the point layer. The search bandwidth was calculated using Silverman’s empirical rule, and the obtained Kernel Density analysis diagram is depicted in Fig 5. The spatial agglomeration characteristics of abandoned cropland varied with location, especially at the junction area of Yetang and Longtian Town, the junction area of Heshui and Huangbei Town, the northwest area of Luofu Town, and the north of Shuikou Town. Based on impacting factors’ data, most abandoned lands were distributed on mountains with higher slopes. 80% and 37% of the abandoned land had slopes >5° and >15°, respectively. In most cases, abandoned cropland was closer to forestland, with 73% and 17% of such lands less <100 m and > 200 m from forest land, respectively. In addition, the fragmentation of abandoned cropland was prominent, with ≈22% of the land having map plots of <1 mu. Based on the cultivation conditions of abandoned farmland, the road convenience and irrigation conditions were generally poor. About 53% of abandoned farmland was >500 m away from rural roads, whereas ≈30% was >500 m away from ditches.

### Influencing factors on cropland abandonment

After standardizing the independent and dependent variables of the logistic regression model, we carried out maximum partial likelihood estimation in SPSS21.0. The results are shown in Table 3.

**Table 3:** Estimation results of impact factor model for cropland abandonment in the study area.

variable	Regression coefficients	Standard deviation	Wald statistics	Degree of freedom	Significance level	*OR value*
**Constant**	-8.1091	0.1630	2476.23	1.00	0.0000	0.0003
**Parcel size**	-5.2945	0.2444	469.48	1.00	0.0000	0.0050
**Shape index**	0.9998	0.0575	302.67	1.00	0.0000	2.7179
**Elevation**	0.2607	0.0671	15.08	1.00	0.0001	0.7705
**Slope**	0.078	0.043	3.239	1.00	0.072	1.0810
**Soil quality**	-4.0078	0.0948	1787.54	1.00	0.0000	0.0182
**Cultivated land output**	-7.1150	0.1397	2595.78	1.00	0.0000	12.3028
**Commuting distance**	0.1799	0.0523	11.85	1.00	0.0006	0.8353
**Distance to woodland**	-0.2231	0.0483	21.34	1.00	0.0000	1.2500
**Distance to ditch**	0.2413	0.0397	36.96	1.00	0.0000	0.7856
**Distance to road**	0.1612	0.0530	9.24	1.00	0.0024	1.1750

From Table 3, we observed that the significance level of the slope was 0.072, i.e., it failed the significance test. The significance level of the remaining nine independent variables was <0.05, indicating significant correlations between the cropland abandonment and the nine independent variables. In addition, the nine independent variables showed different degrees of impact on abandoned farmlands. The influence degrees of the cultivated land and road proximity were the largest and the least, respectively. The contribution of the influencing factors followed the trend: cultivated land output > parcel size > soil quality > shape index > elevation > distance to ditch > distance to woodland > commuting distance > distance to road.

Three farming condition factors viz. commuting distance, distance to ditch, and distance to woodland correlated positively to cultivated land abandonment were identified. Among the six physical geographic factors, cultivated land output evinced the greatest impact (coefficient of -7.1150) on the abandonment. Parcel size was the second most important factor, having an influence coefficient of -5.2945, indicating that the fragmentation of cultivated land in the study area was crucial to cultivated land abandonment. In addition, soil quality and distance to woodland correlated negatively with cultivated land abandonment, indicating that the higher the soil quality and the farther the forest land, the lower the probability of abandoning the cultivated land. Moreover, the negative correlation evinced by the shape index indicates that the higher the elevation, and the more irregular the plot shape, the more likely the land would be abandoned.

### Classification of abandoned cropland

Factor analysis in the SPSS environment was used to cluster the significant factors of cropland abandonment. According to the KMO and Bartlett test, the significance (P-value) was <0.05, rejecting the assumption that each variable was independent. In other words, a strong correlation exists between dependent variable and independent variables. Using the characteristic roots of factors, the significant factors influencing cropland abandonment can be classified into three categories (Table 4) viz. (i) the rotation component matrix, where the first common factor has a larger load factor for commuting distance, distance to road, and distance to ditch. These are farming conditions-driven abandonment; (ii) A larger load factor for soil quality and cultivated land output. These are cultivated land output-driven abandonment; (iii) A larger load factor for land size and land shape, reflecting the influence of cultivated land’s conditions on abandonment; thus, they are land condition-driven abandonment.

**Table 4:** Variable rotation component matrix.

variable	Ingredients		
	1	2	3
**Parcel size**	-0.06	0.05	0.87
**Shape index**	0.41	-0.05	0.67
**Elevation**	0.56	-0.30	-0.09
**Soil quality**	-0.07	0.94	0.01
**Cultivated land output**	-0.11	0.95	-0.02
**Commuting distance**	0.78	0.01	0.13
**Distance to woodland**	-0.30	0.28	0.25
**Distance to ditch**	0.62	-0.12	0.01
**Distance to road**	0.78	0.00	0.14

## Discussion

### The occurrence mechanism of abandoned farmland

Arable lands are abandoned due to multiple factors such as non-agricultural and aging rural labor force. More importantly, it is governed by a development law. From our results, cropland abandonment at plot scale is mainly driven by cultivated land output, tillage conditions, and plot conditions.

The output of cropland is crucial to determining whether cropland was abandoned or not. Since China’s reform and opening up, many rural laborers have relocated to the cities, and educated young people have abandoned agriculture, leaving the elderly, women, and children as the main production force in most rural areas. Therefore, a low labor force has gradually become the main obstacle to agricultural production. However, limited by mountains and hilly terrains, labor-saving machinery is difficult to popularize. Also, limited by labor capacity, farmers must choose to cultivate only a part of the cultivated land. If the output of cropland is low, the efficiency of cultivation will be lower. Because the output obtained from the same labor and capital input is low, this part of the farmland becomes the prioritized abandoned land.

Nevertheless, the worse the farming conditions, the higher the risk of cropland abandonment. From the regression, the distance of farming commuting, irrigation conditions, and traffic convenience in the study area significantly impact the abandonment of cultivated land. The longer the commuting distance of cropland, the more the transportation cost for agricultural inputs. Because of this condition, farmers tend to abandon cultivated land far away from residential areas. Similarly, irrigation of agricultural lands determines the productivity of the farm. Dryland, far from the ditches and with poor irrigation conditions, can only rely on rainfall for watering. This case could also prompt farmers to abandon their farmland. In addition, due to the aging of the farming population, cropland with poor road access cannot be cultivated because agricultural machinery becomes inaccessible and inoperable, leading to the abandonment of farmland.

Finally, land endowments also influence the abandonment of cultivated land significantly. Cultivated land with higher elevation is mainly slopy, therefore, exhibiting poor water and fertilizer retention capacity. Most times, they are drylands, making it difficult to grow rice and other food crops. Due to topographical restrictions, the cost of growing cash crops is also higher. Therefore, slopy land with high elevation often encourages farmers to adopt abandonment. Arable lands close to woodland are also vulnerable to wildlife, therefore making the land susceptible to abandonment. In addition, land fragmentation forms small areas with irregular shapes of cropland, which is not conducive to large-scale and mechanized agriculture. Thence, abandonment of farmland by farmers results.

### Implications for policy

The abandonment of cultivated land is a huge waste of cultivated land resources. Effective control of cropland abandonment is highly significant to ensuring national food security and promoting agricultural and rural development [32]. Presently, cultivated land abandonment of hilly and mountainous areas is relatively severe, needing urgent control measures following local conditions. Due to non-agriculturalization and aging of rural labor, cultivated land abandonment at the plot level is mainly affected by land output, farming conditions, and plot conditions. Therefore, a differentiated governance path should be proposed for different types of abandoned farmlands.

Regarding the impact of low output of cultivated land on abandoned cultivated land, the agricultural structure’s quality and efficiency should be improved while promoting its values under local conditions. Market-oriented planting structure adjustments should be established while developing appealing and high-quality products with peculiar regional characteristics, such as "one village, one product, one county, one industry" [33]. Also, the total income of the cultivated land should be increased. Simultaneously, vigorous promotion of agricultural technologies (such as soil fertilization and organic substitution) must be implemented because of different topography and landform characteristics. Such practice would accelerate the fallow rotation or inverted intercropping of different varieties of crops and improve cultivated land fertility.

To minimize the impact of poor farming conditions on the abandonment, the existing policy support should be strengthened, access roads should be constructed, and water conservation facilities should be provided. Moreover, more emphasis should be placed on improving the construction of agricultural infrastructure through mechanization and efficient irrigation systems.

Given the influence of the fragmentation of cropland and the small area of cropland to abandoning farmlands, land transfer should be accelerated, and adjacent plots and fragmented cropland should be encouraged to be transferred. The transfer (via shareholding) could be to new operating entities, such as professional cooperatives, large growers, or family farms. Such practice would promote the appropriate scale management and efficient use of cropland.

### Methodological limitations

This paper identifies cropland abandonment situations and discusses formation mechanism of cropland abandonment from both macro and micro perspectives under circumstances of identification-classification-management strategy. Combination of microscopic land use difference changes and macroscopic remote sensing differences improved experiment precision and avoids large accuracy error of mountainous and hilly areas caused by single remote sensing identification due to mixed pixels, small investigation scopes and influence of land usage information’s hysteretic nature.

However, results of the sample survey cannot fully reflect the recognition accuracy in experiment, causing by limitations of sampling selection. In addition, recognition of abandoned cultivated land based on the changing law of NDVI has the problem of determining thresholds. For example, some other land types, such as garden land, has low recognition effect when distinguishing from abandoned cropland. What’s more, this article displays some natural factors that affect farmers’ abandonment behavior, but does not consider the influence of the relationship between multiple variables on farmers’ abandonment behavior.

## Conclusions

Due to natural constraints and differences in comparative income between urban and rural areas, the non-agriculturalization of agricultural land and the abandonment of cultivated lands are increasing, resulting in a tremendous waste of land resources. In this paper, land-use change data and NDVI difference were used to identify abandoned farmland. A binary logistic regression model identified the main factors causing cropland abandonment. Further, the principal component analysis classified abandoned farmland types and proposed classification management approaches.

Consequently, this study inferred the following:

1. Cultivated land abandonment in the study area accounts for 16.83% of the total cultivated land. The cases were more severe outside than within the main urban area. In most towns, the abandoned farmland ranged 10%– 20%, with Huangbei town and Luofu town having the largest proportions (>29%).
2. A significant correlation exists between each of the nine variables and cropland abandonment. Cultivated land output and distance to roads showed had the highest and lowest impact, respectively.
3. Finally, we suggest adjusting agricultural structure, strengthening agricultural infrastructure, and constructing new facilities could revamp the abandoned cultivated farmlands in the study area.
